# Differences in the quality of primary medical care for CVD and diabetes across the NHS: evidence from the quality and outcomes framework

**DOI:** 10.1186/1472-6963-7-74

**Published:** 2007-05-29

**Authors:** Gary McLean, Bruce Guthrie, Matt Sutton

**Affiliations:** 1 General Practice & Primary Care, University of Glasgow, 1 Horselethill Road Glasgow, G12 9LX, UK; 2 Tayside Centre for General Practice, University of Dundee, The Mackenzie Building, Kirsty Semple Way, DD2 4BF UK; 3 Health Economics Research Unit, University of Aberdeen, Polworth Building, Fosterhill, Aberdeen, AB25 2ZD, UK

## Abstract

**Background:**

Health policy in the UK has rapidly diverged since devolution in 1999. However, there is relatively little comparative data available to examine the impact of this natural experiment in the four UK countries. The Quality and Outcomes Framework of the 2004 General Medical Services Contract provides a new and potentially rich source of comparable clinical quality data through which we compare quality of primary medical care for coronary heart disease (CHD), stroke, hypertension and diabetes across the four UK countries.

**Methods:**

A cross-sectional analysis was undertaken involving 10,064 general practices in England, Scotland, Wales and Northern Ireland. The main outcome measures were prevalence rates for CHD, stroke, hypertension and diabetes. Achievement on 14 simple process, 3 complex process, 9 intermediate outcome and 5 treatment indicators for the four clinical areas.

**Results:**

Prevalence varies by up to 28% between the four UK countries, which is not reflected in resource distribution between countries, and penalises practices in the high prevalence countries (Wales and Scotland). Differences in simple process measures across countries are small. Larger differences are found for complex process, intermediate outcome and treatment measures, most notably for Wales, which has consistently lower quality of care. Scotland has generally higher quality than England and Northern Ireland is most consistently the highest quality.

**Conclusion:**

Previously identified weaknesses in Wales related to waiting times appear to reflect a more general quality problem within NHS Wales. Identifying explanations for the observed differences is limited by the lack of comparable data on practice resources and organisation. Maximising the value of cross-jurisdictional comparisons of the ongoing natural experiment of health policy divergence within the UK requires more detailed examination of resource and organisational differences.

## Background

Since devolution in 1999, health policy has increasingly diverged in the four UK countries (England, Scotland, Wales and Northern Ireland). [1,2] Health policy across the UK continues to share a number of similar objectives including reducing waiting times, shifting care from hospital to community, reducing health inequalities, and improving clinical quality for people with long term conditions. [3,4] However, the preferred mechanisms for delivering these objectives increasingly vary. [5-8] Post-devolution, England initially focused on centralised performance management, with rewards and sanctions for trusts that succeeded or failed to achieve centrally set targets. Latterly, the emphasis has shifted to implementing a regulated healthcare market built around patient choice and increased plurality of providers, allied to an increasing role for the private sector within the primary care setting. [5-7] Scotland has abolished the purchaser-provider split and (re)created vertically integrated Health Boards, with an emphasis on professionalism and clinical leadership, particularly in managed clinical networks to co-ordinate care across organisational and professional boundaries. [8] The distinctive feature of Welsh policy is a strong emphasis on public health, with local health boards having a duty of partnership with local authorities in commissioning. [9] In Northern Ireland, political uncertainty has meant that major organisational reform has not been implemented. [1]

Therefore a natural experiment is in progress in UK healthcare, and cross-jurisdictional comparison is of growing interest. Examining this natural experiment is difficult because data are often not comparable, partly because data definitions and collection are not consistent, even for healthcare expenditure and analysis of National Health Service (NHS) workforce. [3] Two recent comparisons indicate that England has been more successful than Wales and Northern Ireland in rapidly reducing waiting times for first outpatient appointment and elective surgery, although these are now also falling in Wales and Northern Ireland. Though not fully comparable, Scottish waiting times are consistently lower over the whole period despite waiting times in England falling faster than in Scotland between 2002 and 2005. [3,4,10] Other research has shown that Scotland has higher rates of breast cancer screening and some immunisations, whereas England has lower mortality from colorectal cancer and ischaemic heart disease, and Wales has the highest rate of statin prescribing. [11] There are substantial variations in per-capita healthcare spending, with England spending the least and Scotland the most, although at country level, these differences are partly explained by differences in need, and have reduced post-devolution due to faster growth in healthcare spending in England. [3] Nonetheless, mean listsize per whole time equivalent GP remains lower in Scotland than in other UK countries (partly due to greater rurality). [12] There is no recent comparable data on practice nurse and other practice employed staff available at national levels (Table 1). [13]

**Table 1:** Structural GP and practice characteristics in four UK countries

Variable	England	Wales	Scotland	Northern Ireland
Number of practices	8542	501	1056	366
Number of whole-time equivalent (WTE) GPs	31523	1816	3782	1078
Average registered population	6401	6171	5249	5361
Average number of GPs per practice	3.7	3.6	3.6	3.0
Registered population per WTE GP	1666	1674	1343	1663
Single-partner practices (%)	23	19	16	19
Practices with six or more GPs (%)	21	16	20	9

*All figures for 2004-05 with the exception of Northern Ireland 2003-4[12]

The Quality and Outcomes Framework (QOF) of the 2004 General Medical Services contract provides a new opportunity to compare clinical quality using measures with consistent definition and data collection in all four countries. [3,4,14] However, although QOF is designed to incentivise high quality care and is unique in scale and scope for a national pay-for-performance program, it is not comprehensive in its coverage. There remain a wide variety of measures that can have a significant impact on the quality of care, which are not included. Nevertheless, QOF is consistent across all four UK counties and offers the best current opportunity to compare differences in clinical quality. QOF data is reported at practice level as the proportion of eligible patients achieving each indicator, and case-mix adjustment using patient level data is therefore not possible. However, data reported for payment (which we call 'payment' quality and others have called 'reported' quality [22] allows practices to 'exception report' individual patients for a range of reasons, including patient not attending for care, patient dissent, and patient on maximum treatment. An exception reported patient is removed from the denominator for each indicator. In principle, exception reporting should therefore adjust for case-mix variation. For example, in Scotland in 2004/5, there is no consistent socio-economic gradient in quality using 'payment' quality (which allows exceptions), whereas practices serving more deprived populations have lower 'population' achievement for many measures (calculation of which does not allow exceptions). [15] This paper uses QOF data to compare the quality of care for diabetes and cardio-vascular disease (coronary heart disease [CHD], stroke and transient ischaemic attack, and hypertension) in the four UK countries. All of these conditions cause considerable morbidity and mortality, and the relevant QOF measures are underpinned by a strong evidence base of guidelines and National Service Frameworks. [14] Differences in the average QOF financial incentive in each country are examined by comparing QOF reported prevalence of incentivised disease. QOF payment within countries varies by prevalence of disease in each practice, but this is compared to mean prevalence in each country. However, as payment for the average prevalence practice achieving the same points total in each country is the same, the average per-patient rewards for quality are therefore lower in higher prevalence countries.

## Methods

The analysis used publicly available data on QOF achievement for each practice. [16-19]. Practices with missing data or those with no patients in their denominators were excluded, leaving 8,214 (96%) practices in England, 1023 (98%) in Scotland, 362 (99%) in Northern Ireland and 459 (92%) in Wales.

The Quality Management and Analysis System (QMAS) reports quality measures in the form of 'percentage of eligible patients achieving measure', from which practices are allowed to exception report patients for a variety of reasons ('payment' quality). Although 'payment quality' is therefore effectively a crude form of case-mix adjustment, any differences between countries might reflect differences in exception reporting as well as differences in actual quality of care.

Exception reporting rates are not directly available in QMAS for 2004/5 data, but 'population achievement' (a measure which does not allow exceptions) can be estimated for measures where the non-excepted denominator is the number of patients on the disease register. This method has been outlined in detail elsewhere. [20] In short, we estimate the register size at 31^st ^March using the maximum value of any denominator in the relevant clinical domain to calculate exception rates and population achievement. For the intermediate outcomes measures, this method assumes that unmeasured patients are uncontrolled. In the analysis we report 'payment quality' as the primary outcome, assuming that there is some adjustment for case-mix, but additionally examine differences in 'population' achievement as a form of sensitivity analysis.

Indicators were classified into four groups: simple process; complex process; intermediate outcome (e.g. level of blood pressure achieved); and treatment (e.g. influenza immunisation). We distinguished simple and complex processes in terms of whether they could easily be delivered opportunistically irrespective of whom the patient was seeing. A simple process is therefore one that can be delivered during routine care by *any *doctor or nurse, such as blood pressure measurement or blood taking. A complex process either requires referral to a specialist such as diabetic retinal screening, or is likely to be done only by particular primary care clinicians such as comprehensive diabetic foot examination. Table 2 details the indicators used in each category.

**Table 2:** List of indicators used

Disease area	Indicator definition	**Payment range**
**Simple process**		
CHD 03	Record of smoking status in the previous 15 months	25–90%
Stroke 03	Record of smoking status in the previous 15 months	25–90%
Hypertension 03	Record of smoking status since diagnosis	25–90%
Diabetes 03	Record of smoking status in the previous 15 months	25–90%
CHD 05	Record of blood pressure in previous 15 months	25–90%
Stroke 05	Record of blood pressure in previous 15 months	25–90%
Hypertension 04	Record of blood pressure in previous 9 months	25–90%
Diabetes 11	Record of blood pressure in the previous 15 months	25–90%
CHD 07	Record of total cholesterol in previous 15 months	25–90%
Stroke 07	Record of total cholesterol in previous 15 months	25–90%
Diabetes 16	Record of total cholesterol in previous 15 months	25–90%
Diabetes 02	Record of BMI in previous 15 months	25–90%
Diabetes 05	Record of H1Abc or equivalent in previous 15 months	25–90%
Diabetes 14	Record of serum creatinine testing in the previous 15 months	25–90%
**Complex Process**		
Diabetes 08	Record of retinal screening in the previous 15 months	25–90%
Diabetes 09	Record of peripheral pulse test in the previous 15 months	25–90%
Diabetes 10	Record of neuropathy testing in the previous 15 months	25–90%
**Outcome**		
CHD 06	Blood pressure recorded in previous 15 months ≤ 150/90	25–70%
Stroke 06	Blood pressure recorded in previous 15 months ≤ 150/90	25–70%
Hypertension 05	Blood pressure recorded in previous 15 months ≤ 150/90	25–70%
Diabetes 12	Blood pressure recorded in previous 15 months ≤ 145/85	25–55%
CHD 08	Total cholesterol recorded in previous 15 months ≤ 5 mmol/l	25–60%
Stroke 08	Total cholesterol recorded in previous 15 months ≤ 5 mmol/l	25–60%
Diabetes 17	Total cholesterol recorded in previous 15 months ≤ 5 mmol/l	25–60%
Diabetes 06	HbA1c recorded in previous 15 months ≤ 7.4%	25–55%
Diabetes 07	HbA1c recorded in previous 15 months ≤ 10%	25–85%
**Treatment**		
CHD 09	Aspirin, alternative anti-platelet or anti-coagulant being taken	25–90%
CHD 10	Treated with beta-blocker	25–50%
CHD 12	Record of Influenza immunisation in previous flu season	25–85%
Stroke 12	Record of Influenza immunisation in previous flu season	25–85%
Diabetes 12	Record of Influenza immunisation in previous flu season	25–85%

A simple composite of the achievement levels by indicator category was calculated for each country, by adding up the mean values for each indicator within a particular group and then dividing by the number of indicators in that group (e.g. for complex process indicators- average achievement levels of 70%, 80% and 75% would be added together and divided by three to give a composite achievement level of 75%).

Summary statistics and analyses were based on practice-level data weighted by register size. Since the distributions of many of the quality indicators are non-normal, we provide bootstrapped confidence intervals based on 2,000 replications. [21] We use 99% confidence levels in recognition of the large number of comparisons.

Crude prevalence rates for each disease in each country were calculated for each disease domain by dividing the total number of patients on practice disease registers by the total populations registered with practices. The analysis was undertaken in STATA v8.2.

## Results

Table 3 shows average payment and population achievement in each of the four indicator categories by country. Northern Ireland has the highest achievement under both payment and population achievement for simple process, intermediate outcomes and treatment indicators. Scotland has the highest achievement for the complex process indicators. Wales has the lowest achievement for all categories for both payment and population achievement.

**Table 3:** Average percentage achievement by indicator category and country

Category	England	Scotland	Wales	Northern Ireland
*'Payment quality'*				
Simple (14 measures)	93.4	93.7	92.7	94.4
Complex (3 measures)	80.4	84.5	75.3	81.6
Outcome (9 measures)	72.3	74.7	72.0	76.3
Treatment (5 measures)	82.4	83.4	79.8	85.4
				
*'Population achievement'*				
Simple (14 measures)	91.9	92.6	91.6	93.4
Complex (3 measures)	76.4	79.1	71.9	77.7
Outcome (9 measures)	68.2	69.8	67.1	72.2
Treatment (5 measures)	72.6	72.8	68.3	76.4

Table 4 shows that the higher achievement levels in Northern Ireland compared to England are statistically significant. Scotland has significantly higher achievement than England for all indicator categories under payment quality but not for the treatment measures under population achievement due to higher exclusion rates. Wales has significantly lower achievement than England for complex process and treatment indicators for both payment and population achievement and for outcomes based on population achievement only.

**Table 4:** Average achievement by indicator category for England, and differences from England for other countries

Category	England (%)	Scotland % point difference from England (99% CI)	Wales % point difference from England (99% CI)	Northern Ireland % point difference from England (99% CI)
*'Payment quality'*				
Simple (14 measures)	93.4	**0.3 (0.1 to 0.5**)	-0.7 (-0.9 to 0.2)	**1.0 (0.6 to 1.4)**
Complex (3 measures)	80.4	**4.1 (2.1 to 6.0)**	-**5.1 (-6.4 to 3.7)**	**1.2 (0.7 to 1.8)**
Outcome (9 measures)	72.3	**2.4 (1.8 to 3.2)**	-0.3 (-1.1 to 0.1)	**4.0 (2.1 to 6.0)**
Treatment (5 measures)	82.4	**1.0 (0.4 to 1.5)**	**-2.6 (-3.4 to -1.7)**	**3.0 (2.2 to 3.9)**
				
*'Population achievement'*				
Simple (14 measures)	91.9	**0.7 (0.5 to 0.9)**	-0.2 (-0.5 to 0.1)	**1.5 (1.2 to 1.8)**
Complex (3 measures)	76.4	**2.7 (3.9 to 2.6)**	**-4.5 (-6.0 to -2.9)**	**1.3 (0.2 to 2.4)**
Outcome (9 measures)	68.2	**1.6 (0.7 to 2.5)**	**-1.1 (-1.6 to -0.4)**	**4.0 (3.3 to 4.7)**
Treatment (5 measures)	72.6	0.2 (-0.4 to 0.3)	**-4.3 (5.3 to 3.2)**	**3.8 (2.8 to 4.7)**

Note: Bold type = significant at P < 0.01.

Achievement across the process, intermediate outcome and treatment indicators is generally highest in Northern Ireland and lowest in Wales (Tables 5 and 6). Northern Ireland has significantly higher quality than England for 23 of the 31 indicators and lower quality only for the percentage of diabetics with HbA1c < 7.4. Wales has significantly lower quality than England for 17 of the 31 indicators. Scotland has significantly higher quality than England on 19 indicators and significantly lower quality on 5 indicators.

**Table 5:** Average achievement on each process indicator for England and differences from England for other countries (99% confidence intervals)

**Indicator**	**England (%)**	**Scotland % point difference from England (99% CI)**	**Wales % point difference from England (99% CI)**	**Northern Ireland % point difference from England (99% CI) **
**Simple process measures**				
CHD smoking status recorded	95.0	**0.5 (0.1 to 0.9)**	-0.3 (-1.0 to 0.4)	**1.1 (0.4 to 1.7)**
Stroke smoking status recorded	92.6	**1.5 (0.8 to 2.2)**	-0.6 (-1.8 to 0.7)	**2.0 (1.1 to 3.0)**
BP smoking status recorded	94.2	**1.2 (0.7 to 1.6)**	0.4 (-0.4 to 1.0)	**0.7 (0.1 to 1.3)**
Diabetes smoking status recorded	96.0	**1.4 (1.0 to 1.8)**	-0.4 (-1.1 to 0.2)	**1.3 (0.8 to 1.8)**
				
CHD – blood pressure recorded	96.6	**-1.1 (-1.5 to -0.7)**	-0.2 (-0.7 to 0.2)	0.3 (-0.2 to 0.8)
Stroke – blood pressure recorded	94.9	-0.3 (-0.9 to 2.5)	**-0.9 (-1.9 to -0.0)**	**1.0 (0.2 to 1.6)**
BP – blood pressure recorded	90.2	**-1.3 (-1.9 to -0.7)**	-0.0 (-0.9 to 0.7)	**1.4 (0.5 to 2.3)**
Diabetes – blood pressure recorded	97.1	**0.6 (0.3 to 0.9)**	0.7 (-0.4 to 0.4)	**0.9 (0.5 to 1.2)**
				
CHD – cholesterol recorded	90.3	**-1.4 (-2.3 to -0.5)**	-0.7 (-1.9 to 0.4)	**1.2 (0.1 to 2.3)**
Stroke – cholesterol recorded	84.1	0.9 (-0.3 to 2.3)	**-2.7 (-4.9 to -0.5)**	**3.0 (1.1 to 4.7)**
Diabetes – cholesterol recorded	92.9	**1.5 (0.9 to 2.0)**	-0.1 (-1.1 to 0.8)	**1.8 (1.1 to 2.5)**
				
Diabetes-record of Body Mass Index	90.8	**2.9 (2.4 to 3.3)**	**1.1 (0.4 to 1.8)**	**1.6 (0.7 to 2.4)**
Diabetes-record of HbA1c	94.6	**1.6 (1.2 to 2.0)**	**-0.7 (-1.3 to -0.5)**	**1.8 (1.4 to 2.2)**
Diabetes – creatinine recorded	93.2	**1.3 (0.4 to 1.9)**	-0.7 (-1.7 to 0.2)	0.3 (-0.6 to 1.3)
				
**Complex process measures**				
Diabetes – retinal screening recorded	83.8	**2.7 (1.5 to 3.8)**	**-2.9 (-4.9 to -0.7)**	-0.2 (-2.3 to 0.7)
Diabetes – peripheral pulses recorded	79.4	**5.0 (3.6 to 6.3)**	**-4.8 (-7.6 to -2.3)**	1.9 (-0.8 to 4.3)
Diabetes – neuropathy testing recorded	78.1	**4.6 (3.1 to 6.1)**	**-7.7 (-10.9 to -4.5)**	2.0 (-0.6 to 4.3)

Note: Bold type = significant at P < 0.01

**Table 6:** Average achievement on each outcome and treatment indicator for England and differences from England for other countries (99% confidence intervals)

**Indicator**	**England (%)**	**Scotland % point difference from England (99% CI)**	**Wales % point difference from England (99% CI)**	**Northern Ireland % point difference from England (99% CI) **
**Intermediate outcome**				
CHD – blood pressure ≤ 150/90	84.1	**1.1 (0.4 to 1.8)**	-0.8 (-2.1 to 0.2)	**3.5 (2.7 to 4.4)**
Stroke – blood pressure ≤ 150/90	81.0	**2.2 (1.2 to 3.1)**	**-2.1 (-3.6 to -0.7)**	**4.4 (3.2 to 5.4)**
Hypertension – BP ≤ 150/90	71.4	0.9 (-0.1 to 1.8)	**-1.4 (-2.7 to -0.2)**	**5.0 (3.8 to 6.2)**
Diabetes – blood pressure ≤ 145/85	70.4	**4.3 (3.3 to 5.2)**	**-2.0 (-3.4 to -0.5)**	**6.5 (5.4 to 7.8)**
				
CHD – cholesterol ≤ 5 mmol/l	72.2	-1.5 (-2.7 to -0.4)	-1.8 (-3.5 to -0.2)	-0.4 (-2.2 to 0.4)
Stroke – cholesterol ≤ 5 mmol/l	62.7	**2.4 (0.8 to 3.8)**	**-3.7 (-6.1 to -1.4)**	**2.8 (0.8 to 4.7)**
Diabetes – cholesterol ≤ 5 mmol/l	72.0	**2.4 (1.4 to 3.4)**	0.3 (-1.1 to 1.7)	**3.7 (2.5 to 5.0)**
				
Diabetes – HBA1c ≤ 7.4%	59.0	**-2.1 (-3.1 to -1.1)**	**-1.6 (-2.7 to -0.4)**	**-2.1 (-3.6 to -0.5)**
Diabetes – HBA1c ≤ 10%	89.5	**0.6 (0.0 to 1.2)**	**-1.0 (-2.0 to -0.1)**	**1.1 (0.3 to 1.9)**
				
**Treatment**				
CHD – on aspirin or equivalent	90.2	-0.3 (-0.8 to 0.3)	**-1.5 (-2.5 to -0.8)**	**1.7 (0.9 to 2.4)**
CHD – on beta-blocker	64.9	**4.5 (3.4 to 5.7)**	**-1.8 (-3.4 to -0.2)**	**6.7 (4.9 to 8.6)**
CHD – had influenza vaccination	87.2	-0.4 (-1.4 to 0.6)	**-3.4 (-5.0 to -1.7)**	**1.9 (0.6 to 3.2)**
Stroke – had influenza vaccination	84.0	0.2 (-0.8 to 1.3)	**-4.5 (-6.9 to -2.4)**	1.5 (-0.7 to 3.0)
Diabetes – had influenza vaccination	74.4	**-1.6 (-2.6 to -0.9)**	**-4.3 (-5.6 to -3.0)**	**3.2 (2.0 to 4.4)**

Note: Bold type = significant at P < 0.01

For simple process measures, like the recording of blood pressure or smoking status, absolute differences between the highest and lowest performing countries are generally small and probably of little clinical significance. Most differences are less than 1% and only cholesterol recording in people with stroke shows a difference greater than 5% between Northern Ireland and Wales. For the more complex diabetes process measures, such as retinopathy screening, absolute differences between highest and lowest performing countries are larger (5–10%) and Scotland has the highest quality. For intermediate outcome and treatment indicators, absolute differences are also greater, and clinically significant. Again, Northern Ireland generally has the highest quality and Wales the lowest.

Table 7 shows that average prevalence rates vary by up to 28% (the difference in CHD prevalence between England and Scotland). England has the lowest reported prevalence for CHD and stroke, and Northern Ireland for diabetes and hypertension. Prevalence rates are highest for CHD and stroke in Scotland and for diabetes and hypertension in Wales

**Table 7:** Prevalence rates reported in the Quality and Outcomes Framework in the four UK countries

**Prevalence (%) **	**England**	**Scotland**	**Wales**	**Northern Ireland**
CHD	3.59	4.61	4.20	4.28
Stroke	1.43	1.80	1.69	1.50
Hypertension	11.32	11.85	12.70	10.60
Diabetes	3.45	3.50	3.90	3.00
**Ratio to England**				
CHD	1	1.28	1.17	1.19
Stroke	1	1.26	1.18	1.05
Hypertension	1	1.05	1.12	0.94
Diabetes	1	1.01	1.13	0.87

## Discussion

This is the first study to undertake a comparison of quality of care between the four countries of the UK using QOF data. The key findings are that the quality of care measured by the QOF is generally highest in Northern Ireland and lowest in Wales, and that the financial incentive for quality is proportionately lower in Wales and Scotland. The strength of the study is that it uses clinical quality data for important diseases collected to a common definition for 96% of UK general practices. Although QOF is not a fully comprehensive quality dataset, it exceeds all previous cross-jurisdictional comparisons in terms of the scope and coverage of primary care clinical activity. However, the analysis is limited by the nature of the data, which reflects its origin in a payment, rather than a quality monitoring, system. In particular, the data are at practice level, which makes patient-level case-mix adjustment impossible. Although exception reporting of patients who are unsuitable for or who decline care should function as a crude form of patient level case-mix adjustment, exception reporting is under practice control, potentially serves practices' financial self-interests, and could systematically vary by country. However, the overall conclusions are unchanged whether examining quality measures that allow exception reporting or not. Finally, although QOF data are consistent, other practice level variables are not (for example, data on practice employed staff) which limits exploration of potential resource or organisational explanations for the differences that are observed.

Cross-jurisdictional comparisons using routine data are helpful in identifying broad trends and areas of concern. QOF adds to existing comparisons because of its focus on clinical quality, and its near comprehensive coverage. However, the lack of comparable data on practice resources and organisation means that it is difficult to account for the differences seen beyond generating hypotheses for more detailed examination.

The most notable finding is that quality of CHD, stroke and diabetes care in Wales on these measures is significantly lower than elsewhere in the UK for complex care processes, intermediate outcomes, and treatments. Although varying prevalence between countries means that practices in Wales are, on average, less financially incentivised for quality under QOF, this seems unlikely to have directly influenced motivation to deliver high quality in the first year because the detailed link between performance and reward in the payment system is opaque. [15] However, in the longer term, lower payment rates per patient for the same level of quality might be expected to affect quality. Notably, the measures for which Wales consistently performs poorly are those for which practices are more likely to require collaboration with larger NHS organisations (organisation of diabetes care, effective management of intermediate outcomes, influenza vaccination), rather than simple processes directly under practice control. The evidence from this study is therefore consistent with previously identified waiting time problems being symptomatic of a wider quality problem within NHS Wales, and requires closer examination.

NHS Scotland has generally higher quality than NHS England, particularly on the complex process measures for diabetes. The largest difference is for diabetic foot examination. In theory, Scottish policy on the co-ordination of care for whole populations by managed clinical networks might be expected to have a beneficial impact on such indicators. This finding is also consistent with other evidence of higher quality in Scotland for services requiring area-based co-ordination like immunisation and breast screening. [11] On average, Scotland has substantially more whole time equivalent GPs per 1000 registered patients than England which might also be expected to lead to higher QOF performance (although the higher prevalence of cardiovascular disease implies more work per GP than registered list differences alone imply).

The generally small differences that we have observed may be reassuring for NHS England, where the focus of targets and market reform on acute services and elective surgery does not appear to have seriously affected quality of chronic disease care, at least for these incentivised measures. However, the true test of the success of a policy of increasing market reform and the setting of targets in primary care will require additional research on how the introduction of the QOF has impacted on those areas not covered by the GMS contract.

Finally, it is striking that clinical quality in Northern Ireland is higher than in the rest of the UK. This may reflect greater population or health service stability, for example because Northern Ireland has experienced less diversion of management and clinical attention due to repeated re-organisation. It may also in part be a consequence of Northern Ireland having a younger population compared to the UK as a whole with evidence from England suggesting that practices with a higher proportion of over 65 year-olds have lower achievement. [22]

## Conclusion

Previously identified weaknesses in Wales related to waiting times appear to reflect a more general quality problem within NHS Wales. The introduction of QOF data and this subsequent analysis adds to the existing relatively sparse literature on cross-jurisdictional differences in quality of care within the UK. However, such routine data sets are predominately useful for identifying overall patterns and areas for more detailed examination by focused research projects. Although some research to tease out the impact of diverging health policy has recently been funded, [23,24] maximising the potential for cross-jurisdictional learning within the UK will require more detailed, comparative examination of the organisation and resourcing of primary medical care, and collection of a wider range of clinical process and outcome measures, including care for non-incentivised diseases.

## Competing interests

The author(s) declare that they have no competing interests.

## Contributorship

BG had the original idea, and planned the analysis with GM and MS. GM conducted the data analysis and all three authors wrote and revised the manuscript. GM is the guarantor.

## Pre-publication history

The pre-publication history for this paper can be accessed here:


